# Use of the 23S rRNA gene as a target template in the universal loop-mediated isothermal amplification (LAMP) of genomic DNA from phytoplasmas

**DOI:** 10.1128/spectrum.00106-24

**Published:** 2024-03-27

**Authors:** Mako Akahori, Akio Miyazaki, Hiroaki Koinuma, Ryosuke Tokuda, Nozomu Iwabuchi, Yugo Kitazawa, Kensaku Maejima, Shigetou Namba, Yasuyuki Yamaji

**Affiliations:** 1 Department of Agricultural and Environmental Biology, Graduate School of Agricultural and Life Sciences, The University of Tokyo, Bunkyo-ku, Tokyo, Japan; USDA - San Joaquin Valley Agricultural Sciences Center, Parlier, California, USA

**Keywords:** CTAB, FTA card, on-site detection, ribosomal RNA operon, simple DNA extraction, insect vector

## Abstract

Plant-pathogenic bacteria cause numerous diseases in host plants and can result in serious damage. Timely and accurate diagnostic techniques are, therefore, crucial. While advances in molecular techniques have led to diagnostic systems able to distinguish known plant pathogens at the species or strain level, systems covering larger categories are mostly lacking. In this study, a specific and universal LAMP-based diagnostic system was developed for phytoplasmas, a large group of insect-borne plant-pathogenic bacteria that cause significant agricultural losses worldwide. Targeting the 23S rRNA gene of phytoplasma, the newly designed primer set CaPU23S-4 detected 31 ‘*Candidatus* Phytoplasma’ tested within 30 min. This primer set also showed high specificity, without false-positive results for other bacteria (including close relatives of phytoplasmas) or healthy plants. The detection sensitivity was ~10,000 times higher than that of PCR methods for phytoplasma detection. A simple, rapid method of DNA extraction, by boiling phytoplasma-infected tissues, was developed as well. When used together with the universal LAMP assay, it enabled the prompt and accurate detection of phytoplasmas from plants and insects. The results demonstrate the potential of the 23S rRNA gene as a versatile target for the LAMP-based universal detection of bacteria at the genus level and provide a novel avenue for exploring this gene as molecular marker for phytoplasma presence detection.

**IMPORTANCE:**

Phytoplasmas are associated with economically important diseases in crops worldwide, including lethal yellowing of coconut palm, “flavescence dorée” and “bois noir” of grapevine, X-disease in stone fruits, and white leaf and grassy shoot in sugarcane. Numerous LAMP-based diagnostic assays, mostly targeting the 16S rRNA gene, have been reported for phytoplasmas. However, these assays can only detect a limited number of ‘*Candidatus* Phytoplasma’ species, whereas the genus includes at least 50 of these species. In this study, a universal, specific, and rapid diagnostic system was developed that can detect all provisionally classified phytoplasmas within 1 h by combining the LAMP technique targeting the 23S rRNA gene with a simple method for DNA extraction. This diagnostic system will facilitate the on-site detection of phytoplasmas and may aid in the discovery of new phytoplasma-associated diseases and putative insect vectors, irrespective of the availability of infrastructure and experimental resources.

## INTRODUCTION

The spread of various diseases in humans, animals, and plants have caused tremendous social and economic damage (1, 2). In terms of agricultural production, large-scale infections by plant-pathogenic bacteria pose a threat to healthy plant growth, with the potential to cause significant economic losses worldwide, despite efforts aimed at disease control and reduction (3, 4). Plant-pathogenic bacteria of agricultural importance include members of the genera *Pseudomonas*, *Ralstonia*, *Agrobacterium*, *Xanthomonas*, *Erwinia*, *Xylella*, *Dickeya*, *Pectobacterium*, *Clavibacter*, and ‘*Candidatus* Phytoplasma’ (5, 6). Among the symptoms induced by these bacterial infections are galls, overgrowths, wilts, leaf spots, specks, blights, soft rots, scabs, and cankers (5). Due to the difficulty controlling these pathogens, their timely and accurate detection is crucial to minimize yield losses.

Many molecular detection techniques are available to detect a narrow range of plant-pathogenic bacteria, either a specific species or specific strains within a species (7), whereas universal methods allowing the detection of a broad range of bacterial pathogens, such as the bacterial genus described above with similar epidemiological characteristics and common control measures, are lacking. Closely related bacterial species usually cause similar diseases on a large variety of plants, with the detection of all of those species thus far requiring a large number of specific detection assays. A universal detection system, by contrast, would allow the rapid and simple detection of those species, in addition to significantly reducing labor, costs, and resources.

Phytoplasmas are a large group of phloem-limited, plant-pathogenic bacteria belonging to the genus ‘*Candidatus* Phytoplasma’ (8) consisting of at least 50 phytopathogenic species. They infect more than 1,000 plant species and are transmitted by phloem-feeding insect vectors mainly belonging to the order Hemiptera (9, 10). Plant diseases associated with phytoplasmas pose a major threat to the agricultural production worldwide (11), especially in developing countries, where infrastructure and experimental resources are often inadequate. Among the diseases induced by phytoplasmas are lethal yellowing of coconut palm, “flavescence dorée” and “bois noir” of grapevine, apple proliferation disease, X-disease of stone fruits, frog skin of cassava, white leaf of sugarcane, yellows of canola, yellow dwarf disease of rice, and phyllody of sesame (11–15). Many of the symptoms of phytoplasma infection are often confused with viral symptoms (15); in fact, until half a century ago, phytoplasma diseases were believed to be caused by viruses (11). Currently, control measures for phytoplasma diseases are limited to insect vector control and elimination of infected plants, as no practical bactericides or resistant varieties have been reported (11, 15, 16). On-site diagnostic techniques are, therefore, important for timely phytoplasma disease control.

Ribosomal RNA genes (rDNAs), including 5S, 16S, and 23S rDNAs, are highly conserved housekeeping genes and in prokaryotes they have been used for classification (17–21). Although phytoplasmas were first recognized as mycoplasma-like organisms under electron microscopy at the time of their discovery (22), phylogenetic analysis using 16S rDNA grouped them as a newly designated genus ‘*Ca*. Phytoplasma’, distinct from animal mycoplasmas (8). ‘*Ca*. Phytoplasma’ share two copies of the ribosomal RNA operon (23, 24), and species classification using 16S rDNA identity has thus far revealed 50 species (8, 13, 25). For convenience, these species have been broadly divided into eight 16S-groups (i–viii) based on their phylogenetic relationships of 16S rDNA (26, 27) or into 31 16Sr-RFLP-groups based on restriction fragment length polymorphism of 16S rDNA (28). Numerous 16S rDNA sequences of phytoplasmas have been deposited in online databases. By contrast, 23S rDNA has rarely been used for either classification or the universal detection of phytoplasmas (29, 30).

Phytoplasma diagnostic assays based on LAMP, an isothermal DNA amplification method that enables ultrasensitive, rapid, simple, and cost-effective detection of pathogens of plants and animals (31, 32), are currently limited largely to the 16S rDNA (33) of a specific or a limited number of species. While several universal phytoplasma-detection techniques based on polymerase chain reaction (PCR) and quantitative PCR (qPCR) targeting 16S or 23S rDNA have been reported (30, 34–37), there is no LAMP assay that can detect the entire genus ‘*Ca*. Phytoplasma’ on site, either specifically or comprehensively.

The 23S rDNA has high potential as a target for LAMP primer design, due to its length (ca. 2,900 bp), which is about twice that of 16S rDNA (ca. 1,500 bp). Moreover, 23S rDNA sequence information is accumulating, in particular due to progress in the whole-genome sequencing of phytoplasma. In this study, the 23S rDNA sequence information was used to generate LAMP primers for universal phytoplasma detection. Four candidate LAMP primer sets targeting 23S rDNA were designed, and their performance was assessed using various phytoplasma-infected samples. One of these primer sets was able to detect 31 ‘*Ca*. Phytoplasma’ species without false-positives for healthy plants or other bacteria, including those closely related to phytoplasma. The detection sensitivity of the developed LAMP was far superior to that of PCR. In addition, a simple and rapid DNA extraction method that can be used in combination with the LAMP assay for the on-site detection of phytoplasmas was developed. Protocols for different sample types are described as well.

## RESULTS

### Designing LAMP primer sets for the universal detection of phytoplasmas based on the 23S rDNA region

The aim was to design universal LAMP primer sets that enable the detection of ‘*Ca*. Phytoplasm’ species based on their 23S rDNA sequences. Therefore, to cover the sequence diversity of phytoplasmas, the 16S-group (26, 27), which broadly divides the 50 ‘*Ca*. Phytoplasma’ species into eight groups, was referred to for convenience. Eight 23S rDNA sequences corresponding to each of these 16S-groups of phytoplasmas were obtained from the NCBI database: ‘*Ca*. P. asteris’ (NC005303), ‘*Ca*. P. mali’ (CU469464), ‘*Ca*. P. aurantifolia’ (JQ359013), ‘*Ca*. P. pruni’ (AF277076), ‘*Ca*. P. phoenicium’ (HM559253), ‘*Ca*. P. palmae’ (HQ613875), ‘*Ca*. P. oryzae’ (CP116038), and ‘*Ca*. P. ziziphi’ (GU723425) (Fig. S1).

Real-time LAMP assays were conducted using the four primer sets (Table 1; Table S1) at the optimal temperature for each one and 10 ng DNA from plants infected with each of eight ‘*Ca*. Phytoplasma’ species (‘*Ca*. P. asteris’, ‘*Ca*. P. mali’, ‘*Ca*. P. aurantifolia’, ‘*Ca*. P. pruni’, ‘*Ca*. P. phoenicium’, ‘*Ca*. P. noviguineense’, ‘*Ca*. P. oryzae’, and ‘*Ca*. P. ziziphi’) covering eight 16S-groups (Table S2; Fig. S2). In the LAMP assay, no amplicons using CaPU23S-1 were obtained for any of the eight species except ‘*Ca*. P. asteris’ (Fig. S3a). Similarly, CaPU23S-2 and CaPU23S-3 failed to amplify ‘*Ca*. P. phoenicium’ (Fig. S3b and c). By contrast, the LAMP assay using CaPU23S-4, designed to correspond to the 3' end of 23S rDNA, amplified all eight species within 30 min. Additionally, no amplification was detected for the three negative controls (Fig. S3d). Therefore, due to its superior performance in both universality and detection speed, CaPU23S-4 was selected for further validation.

**TABLE 1:** LAMP primer sequences used in the universal detection of phytoplasmas

Primer name	Sequence (5'−3')
CaPU23S-4-F3	GATGTCGGCTCATCGCAT
CaPU23S-4-B3	CATAGCTACTCAGCTGTGGC
CaPU23S-4-FIP	CTGAACCCAGCTCGCGTTCCCCTGGAGCTGGAGAAGGT
CaPU23S-4-BIP	CCGTCGTGGGCGTTGGAAATTAACTGAAACACCAGCGGTATA
CaPU23S-4-LF	AACAGCCCAACCCTTGGA
CaPU23S-4-LB	GAACTGTCCCTAGTATGAGAAGACC

### Sequence conservation of the CaPU23S-4 region in the genus ‘*Ca*. Phytoplasma’

**Fig 1:**
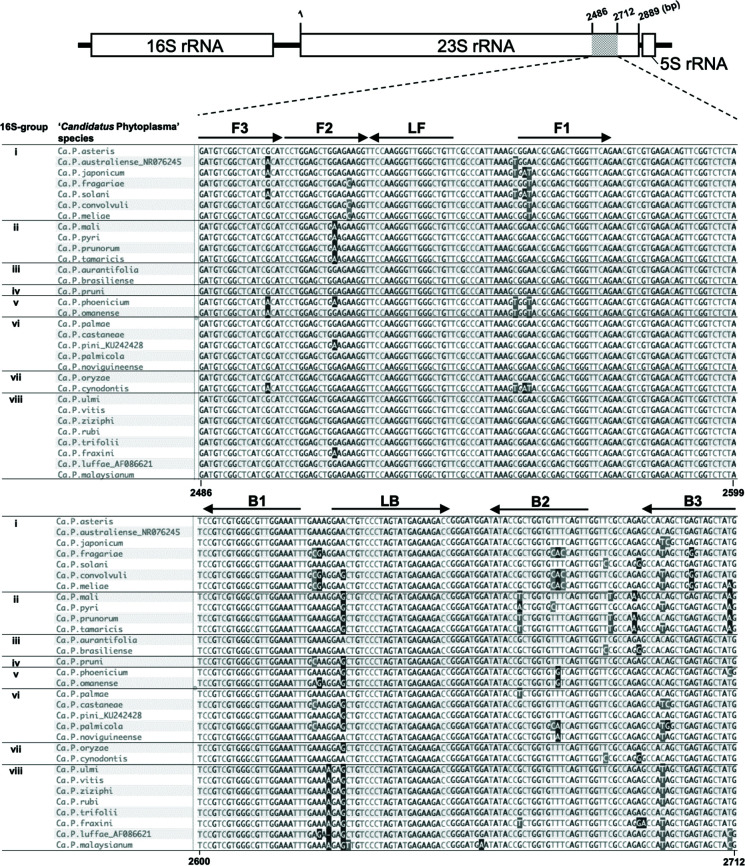
Alignment of the CaPU23S-4 target region in 31 ‘*Candidatus* Phytoplasma’ species. Previously reported phylogenetic groups (16S-groups i–viii) are shown in the left column (26, 27). Artificially synthesized DNAs corresponding to the 23S rDNA sequences of ‘*Ca*. P. australiense’, ‘*Ca*. P. pini’, and ‘*Ca*. P. luffae’ are shown with their DDBJ/GenBank/ EMBL accession numbers. The accession numbers of the other species are shown in Table S2. The position and direction of the CaPU23S-4 primers designed in this study are indicated by arrows. Nucleotides that differ from the primers are highlighted.

To investigate conservation of the region targeted by CaPU23S-4 within the genus ‘*Ca*. Phytoplasma’, the sequences of this region in total 31 ‘*Ca*. Phytoplasma’ species, corresponding to each of the eight 16S-groups, were aligned and compared (Fig. 1; Table S2). For the 28 ‘*Ca*. Phytoplasma’ species for which DNA samples were available, the target sequences were obtained by PCR amplification (Fig. S4), and for the remaining three ‘*Ca*. Phytoplasma’ species (‘*Ca*. P. australiense’, ‘*Ca*. P. pini’, and ‘*Ca*. P. luffae’), sequence data on the NCBI database were obtained. The analysis showed that the region of interest is highly conserved in the genus. The 23S rDNA sequences of ‘*Ca*. P. asteris’ and ‘*Ca*. P. aurantifolia’ perfectly matched CaPU23S-4, while the mismatches between the 23S rDNAs of the other 29 ‘*Ca*. Phytoplasma’ species and CaPU23S-4 were negligible. Moreover, in no case was there a mismatch with CaPU23S-4 at the 3' end region of each primer, which is important for DNA extension, except for a mismatch at the 3' end region of B3 in ‘*Ca*. P. fraxini’.

### Validation of CaPU23S-4 for the universal detection of phytoplasmas by LAMP amplification

**Fig 2:**
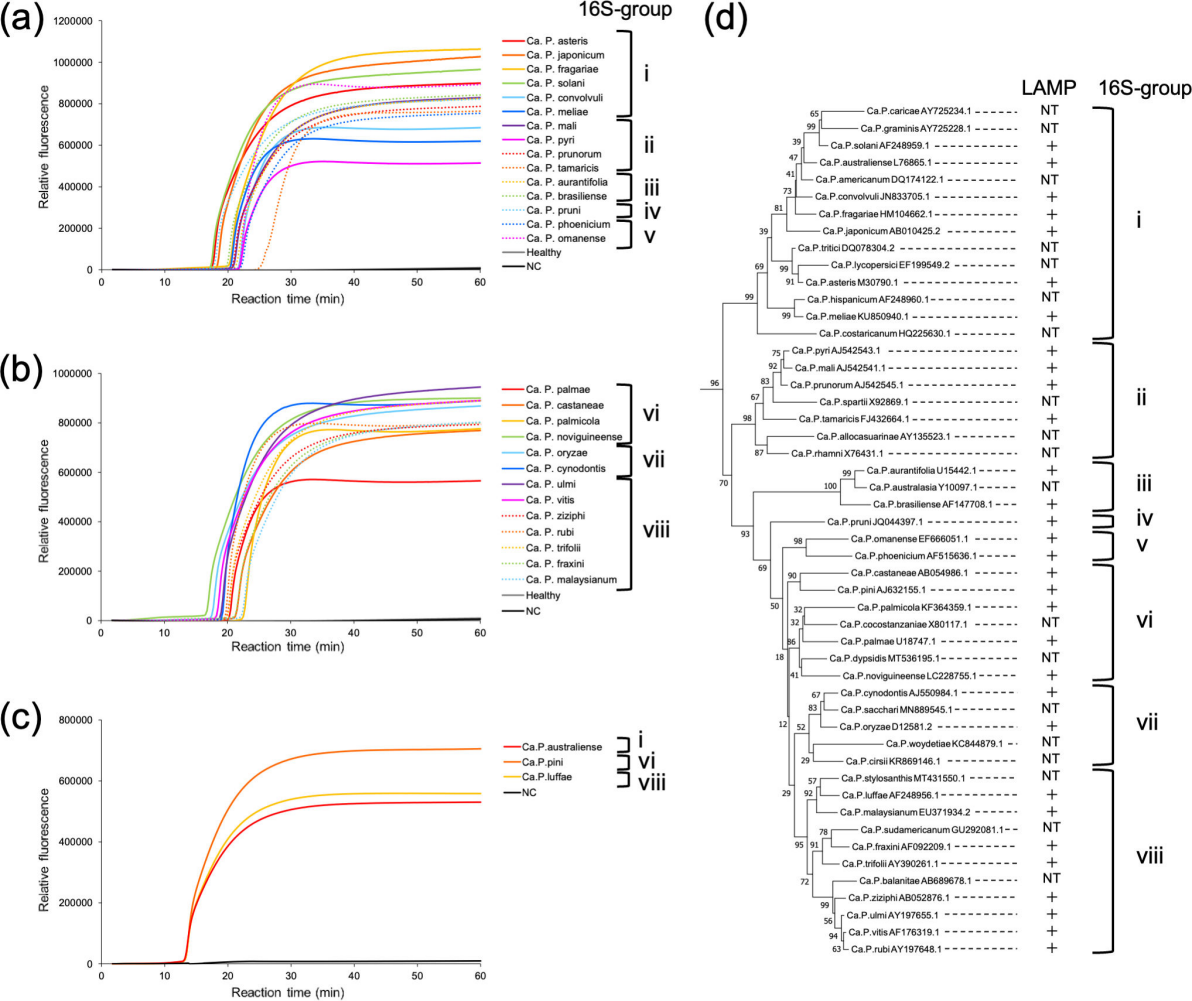
Phytoplasma detection range in the universal LAMP assay. (a–c) LAMP amplification curves obtained with the CaPU23S-4 primer set. LAMP assays were performed using 31 species of phytoplasmas. The LAMP results obtained with DNA (10 ng) from 16S-groups i–v (a) and vi–viii (b) and with the artificially synthesized DNA (10 ng) based on three species (‘*Ca*. P. australiense’, ‘*Ca*. P. pini’, ‘*Ca*. P. luffae’) are shown. LAMP reactions were performed at 64°C for 60 min. As templates for the negative controls, total DNA (10 ng) from a healthy *Glebionis coronaria* (Healthy) and distilled water (NC) were used. (d) Phylogenetic tree based on the16S rDNA of the tested phytoplasma species with the summary of the LAMP assays using CaPU23S-4. The phylogenetic tree was constructed with *Acholeplasma laidlawii* BN1-JA1 (JN935888) and *Bacillus subtilis* (NR112116) as outgroups. Numbers on branches are the percentage of bootstrap values obtained for 500 replicates. LAMP-positive cases are indicated by +, and untested cases are indicated by NT. Note that there is no minus sign (−) indicating LAMP-negative cases since all phytoplasmas tested were LAMP-positive. Eight 16S-groups (i–viii) are shown in the right column (26, 27).

To evaluate the universal detection of phytoplasmas using CaPU23S-4, LAMP assays were conducted that included plants infected with each of 28 ‘*Ca*. Phytoplasma’ species (Table S2). CaPU23S-4 detected ‘*Ca*. P. tamaricis’ in approximately 30 min and all other species, including ‘*Ca*. P. fraxini’, in 15–25 min (Fig. 2a and b). When artificially synthesized DNAs based on the 23S rDNA sequences of ‘*Ca*. P. australiense’, ‘*Ca*. P. pini’, and ‘*Ca*. P. luffae’ were used the assays, all three templates were detected in 15 min (Fig. 2c). The phylogenetic positions and LAMP results of the 31 ‘*Ca*. Phytoplasma’ species tested were shown in a 16S rDNA-based neighbor-joining tree (Fig. 2d; Table S3). These phytoplasmas covered not only the eight 16S-groups but also most of the subclusters in each 16S-group (Fig. 2d). Furthermore, the phytoplasmas detected in this study corresponded to 22 16Sr-RFLP-groups, while the LAMP primers developed in previous studies detected up to eight 16Sr-RFLP-groups (Table S4).

The detection times for each species ranged from 20 to 25 min, with the exception of ~30 min for ‘*Ca*. P. tamaricis’ and ~15 min for the three artificially synthesized DNAs of ‘*Ca*. P. australiense’, ‘*Ca*. P. pini’, and ‘*Ca*. P. luffae’ (Fig. 2a through c). For the 23S rDNA of ‘*Ca*. P. tamaricis’, mismatches with the primer sequences were found in the annealing regions of the primers F2, LB, B2, and B3 of CaPU23S-4 (Fig. 1). However, these were not specific to ‘*Ca*. P. tamaricis’; they were common to some 16S-group ii phytoplasmas. In the case of ‘*Ca*. P. pyri’, which had a larger number of base mismatches on B2 than ‘*Ca*. P. tamaricis’, LAMP amplification required ~25 min (Fig. 1 and 2a), suggesting that the 5 min delay in the detection time of ‘*Ca*. P. tamaricis’ was not due to mismatches with the primer sequences but due to the low phytoplasma titer in the total DNA sample of the infected plant. This conclusion is supported by the fewer amplicons obtained with PCR (23SP-1pf/23SP-1pr) amplification using 10 ng DNA from the ‘*Ca*. P. tamaricis’-infected sample than that of other samples using the same amount of DNA (Fig. S4a). It should be noted that artificially synthesized DNAs are composed of short fragments solely including the target region of the LAMP assay, which results in a higher copy number of templates in these samples than in the same amount of total DNAs from infected plants, thus leading to a faster detection time.

### Specificity of CaPU23S-4 for phytoplasma detection

To evaluate the specificity of CaPU23S-4 for phytoplasma detection, LAMP assays were conducted using purified genomic DNAs from 14 bacterial species, including those closely related to phytoplasmas, such as *Bacillus* (phylum Firmicutes), *Mycoplasma*, *Mesoplasma*, *Ureaplasma*, *Spiroplasma*, and *Acholeplasma* (class Mollicutes) (Table S5). No LAMP amplification was observed for any of the bacterial species at 60 min, which is the typical duration recommended in LAMP detection protocols, or even after 90 min, with the exception of the amplification after 70 min of one of the four replicates of *Acholeplasma*, which is the closest relative of phytoplasmas among the class Mollicutes. The high number of base mismatches for some primers of CaPU23S-4, including positions near the 3' end, is consistent with this result (Fig. S1). LAMP assays were also performed using total DNA extracted from 16 healthy plant species reported as hosts of phytoplasmas (Table S6). The results showed no LAMP amplification in any of the tested species at 90 min.

### Detection sensitivity of CaPU23S-4

**Fig 3:**
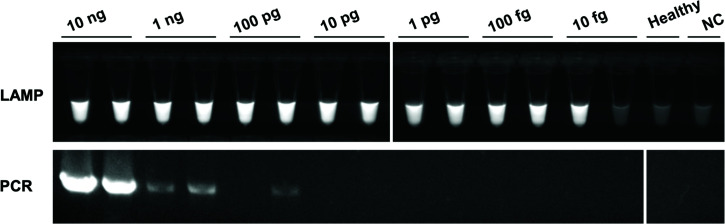
Comparison of the detection sensitivity between the CaPU23S-4 LAMP assay and a universal PCR (P1/P7). The LAMP and PCR assays were performed using a 10-fold dilution series (10 ng to 10 fg per assay) of total DNA extracted from ‘*Ca*. P. asteris’-infected *G. coronaria*. The LAMP reaction was performed for 60 min at 64°C. Healthy: negative control using total DNA (10 ng) of healthy *G. coronaria* as a template; NC, negative control using distilled water.

The detection sensitivity of the LAMP primer CaPU23S-4 was compared to that of universal PCR primers (P1/P7). LAMP and PCR assays were performed against a 10-fold dilution series (ranging from 10 ng to 10 fg per assay) of total DNA extracted from a plant infected with ‘*Ca*. P. asteris’. The detection limit of the universal PCR was determined to be 1 ng, while that of CaPU23S-4 in the LAMP assay was 1 pg or 100 fg, resulting in 1,000- to 10,000-fold higher sensitivity (Fig. 3). Moreover, the detection time for 100 fg total DNA, the detection limit of this LAMP assay, was approximately 25–30 min (Fig. S5).

### Development of a simple DNA extraction method for LAMP assays

**Fig 4:**
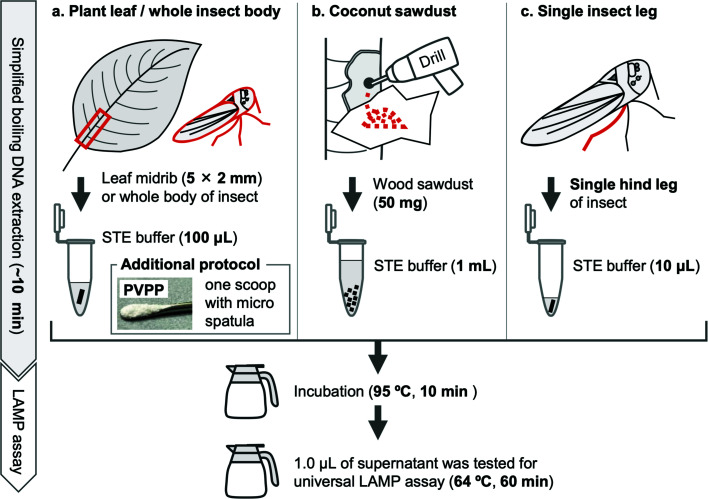
Simplified diagnostic protocol for phytoplasmas using the CaPU23S-4 LAMP assay. DNA was extracted from plant leaf or whole insect body (a), coconut sawdust (b), and a single insect leg (c) using a simple boiling extraction method, achieved by soaking samples in STE buffer, followed by incubation at 95°C for 10 min. Then 1 µL supernatant was used for the template for LAMP reaction. (a) The basic boiling DNA extraction protocol. The leaf midrib (5 × 2 mm) or the whole body of the insect is soaked into 100 µL of STE buffer. As an additional protocol, adding one scoop of polyvinylpolypyrrolidone (PVPP) using a micro-spatula to 100 µL STE buffer can improve DNA extraction. (b) Boiling DNA extraction from wood sawdust. For plants with low concentrations of phytoplasma in the leaves, such as coconut palm, wood sawdust from the trunk was used in the boiling extraction, with 50 mg sawdust per 1 mL STE buffer as the optimal ratio. (c) Boiling DNA extraction from single insect leg. Phytoplasma infection in insects can be tested by boiling DNA extraction from a single hind leg in 10 µL STE buffer.

In the field, sample types and locations vary greatly, and thus to develop a universal, specific, and sensitive test for the detection of phytoplasmas, it is necessary to consider a wide range of DNA-extraction methods and develop optimal protocols for each sample type. There were, therefore, tested three DNA-extraction methods using phytoplasma-infected and noninfected samples from seven host plant species and from an insect vector (Table 2). The three extraction methods were CTAB with purification, the FTA card method, and a newly developed boiling-extraction method (see Materials and Methods), in which DNA is extracted by incubating samples in 100 µL sodium chloride-Tris-EDTA (STE) buffer at 95°C (Fig. 4A). The approximate time required for DNA extraction was 3–4 h for the CTAB method, ~1.5 h for the FTA card method, and ~10 min for the boiling method.

**TABLE 2:** The results of the universal LAMP assay obtained using different DNA extraction methods^*e*^

	Sample species and name^*a*^		Phytoplasma infection (‘Candidatus phytoplasma’ species)		DNA extraction method			
					Boiling		CTAB or purified DNA	FTA card
Plant	*Catharanthus roseus*	Periwinkle	Healthy		−		−	−
			Infected	(‘Ca. P. aurantifolia’)	+		+	+
	*Chrysanthemum coronarium*	Chrysanthemum	Healthy		−		−	−
			Infected	(‘Ca. P. asteris’)	+		+	+
	*Musa* sp.	Banana	Healthy		−		−	−
			Infected	(‘Ca. P. noviguineense’)	+		+	+
	*Hydrangea macrophylla*	Hydrangea	Healthy		−		−	−
			Infected	(‘Ca. P. japonicum’)	+		+	+
	*Manihot esculenta*	Cassava	Healthy		−		−	−
			Infected	(‘Ca. P. pruni’)	+		+	+
	*Elaeocarpus zollingeri*	Elaeocarpus	Healthy		−^*b*^		−	−
			Infected	(‘Ca. P. malaysianum’)	+^*b*^		+	+
	*Cocos nucifera*	Coconut palm	Healthy		−^*c*^		−^*c*^	NT
			Infected	(‘Ca. P. noviguineense’)	+^*c*^		+^*c*^	NT
Insect	*Macrosteles striifrons*		Healthy		−	(−^*d*^)	−	−
			Infected	(‘Ca. P. asteris’)	+	(+^*d*^)	+	+

^*a*^ *C. roseus* and *G. coronaria* are experimental plants, the other plants are field plants and *M. striifrons* is laboratory-maintained insect.

^*b*^ Results obtained with the addition of PVPP to STE buffer.

^*c*^ DNA was extracted from the wood sawdust of *C. nucifera* trunk.

^*d*^ DNA was extracted from a single hind leg of *M. striifrons.*

^*e*^ NT: not tested.

Phytoplasma-infected or noninfected samples from seven host plants (*Glebionis coronaria*, *Cocos nucifera*, *Catharanthus roseus*, *Elaeocarpus zollingeri*, *Hydrangea macrophylla*, *Manihot esculenta*, and *Musa* sp.) and the insect vector (*Macrosteles striifrons*) of the onion yellows (OY) strain of ‘*Ca*. P. asteris’ were tested (Table 2). Leaf midrib is a common sample type used for phytoplasma detection. However, in coconut palm (*C. nucifera*), trunk sawdust is commonly used for phytoplasma detection (38) since the trunk is easier to collect than the leaves at the top of tall trees and, furthermore, the phytoplasma concentration is significantly higher in the trunk than in the leaves (39). For accurate diagnosis, the amounts of sawdust and STE buffer were optimized. Sawdust to STE buffer ratios of 10 mg/100 µL, 50 mg/1 mL, 10 mg/1 mL, and 5 mg/1 mL were tested, determining that 50 mg of sawdust per 1 mL STE buffer was the optimal ratio for LAMP amplification without false negatives (Table S7). Therefore, 50 mg trunk sawdust of *C. nucifera*, the leaf midribs (5 × 2 mm) of the other plants, and the crushed entire body of *M. striifrons* were used as samples (Fig. 4).

Phytoplasmas were successfully detected in six of the seven infected-plant species (*G. coronaria*, *C. nucifera*, *C. roseus*, *H. macrophylla*, *M. esculenta*, and *Musa* sp.) and in the infected-vector insect by all extraction methods (Table 2; Fig. S6). However, the simple boiling extraction method failed to produce LAMP amplification from infected *E. zollingeri* sample (Fig. S6b) in contrast to the positive results obtained from CTAB and FTA card methods. Fluorescence was not detected in any of the noninfected samples, regardless of the DNA-extraction method employed. Woody plants contain high levels of polysaccharides and polyphenols, which can inhibit nucleic acid amplification, such that the removal of these components prior to DNA extraction is recommended (40). Polyvinylpolypyrrolidone (PVPP) facilitates DNA extraction from woody plants, as it removes plant-derived polyphenols, which form complexes with nucleic acids and thereby hinder their amplification (40, 41). Thus, the protocol was modified by adding PVPP to the STE buffer before boiling (Fig. 4A). This led to the successful detection of phytoplasma infecting *E. zollingeri* (Table 2; Fig. S6b). No false-positives due to the addition of PVPP were identified in the noninfected samples.

Finally, the presence of phytoplasmas was examined in the insect whether it could be detected in samples consisting of a single hind leg. DNA was extracted from a dissected single hind leg (with hemolymph) and alimentary canal (as infected control) from each insect after boiling in 10 µL STE buffer (Fig. 4C). Individuals positive for LAMP amplification when using the alimentary canal were also positive when using just a single hind leg (Table 2), demonstrating the validity of using a single hind leg for phytoplasma detection in insect vectors. LAMP amplification was not detected in noninfected samples.

## DISCUSSION

A LAMP-based simple and rapid system for detecting phytoplasmas in various hosts was designed. The CaPU23S-4 primer set exhibited high detection versatility and sensitivity for the genus ‘*Ca*. Phytoplasma’, making it a promising tool for this purpose. In addition, it includes a fast and convenient method of DNA extraction by boiling. This study is the first to report a universal LAMP assay for the accurate, sensitive, simple, and rapid detection of phytoplasmas, thus demonstrating the potential of 23S rDNA as a LAMP assay target for the genus-level identification of these bacteria. This system will significantly improve the on-site detection of phytoplasmas and may facilitate the discovery of new phytoplasma diseases as well as previously unrecognized potential insect vectors, irrespective of the availability of infrastructure and experimental resources.

The key factor contributing to the successful development of this LAMP assay was the use of 23S rDNA. The design requirements for LAMP primers are more stringent than those for PCR or qPCR, as the LAMP method requires the use of six specific primers, including a loop primer that promotes amplification (31). Other considerations include the distance between each primer and the melting temperature (Tm). Because 23S rDNA is approximately twice as long as 16S rDNA, it offers a larger pool of potential target sites for LAMP primers. This difference allowed to obtain a primer set for use in a universal LAMP assay for the genus ‘*Ca*. Phytoplasma’. These results imply that 23S rDNA is a suitable target for LAMP-based universal detection systems, in addition to 16S rDNA, that has been used as the gold standard target for phytoplasma detection.

The specificity and sensitivity of the CaPU23S-4 LAMP primer set make it suitable for the accurate detection of phytoplasmas in practical applications, as the assay avoids the false-positive amplification of closely related bacterial species or host plants and false negatives due to a lack of sensitivity. Surface flora and endophytic bacteria present on and within plants (42, 43) were also not detected by CaPU23S-4. This is contrast to some cases where PCR using P1/P7 or R16F2n/R16R2 amplified plant chloroplast DNA (44, 45) and *Bacillus* DNA (46). This LAMP method is, thus, more specific for phytoplasmas than the PCR method. In addition, the assay can detect phytoplasmas even in samples with titers too low to be detected by PCR. Moreover, the detection time of this method at the lower limit of detection (100 fg) was about 25–30 min (Fig. S5). These findings demonstrate the applicability of CaPU23S-4 as a highly reliable LAMP primer set for the detection of phytoplasmas.

Several DNA extraction methods have been used for the on-site detection of phytoplasmas, including strategies based on lateral flow devices and the grinding of plant tissue in Tris-EDTA-Triton X-100 buffer combined with the LAMP reaction (47–50). The boiling method developed in this study is short in extraction time, simple in operation, directly applicable to LAMP assays, and generates little waste. Furthermore, the method was applicable to a wide variety of sample types, indicating its potential contribution to on-site diagnosis of a wide range of phytoplasma diseases. In a previous study, infection checks of coconut palms were conducted by soaking the wood sawdust of the trees in CTAB extraction buffer for 48 h before DNA extraction using the phenol-chloroform method and alcohol precipitation (38). However, with the boiling method, DNA extraction was complete in just 10 min, a significant time savings that allows a faster diagnosis. Further testing of the applicability of DNA extraction by boiling in additional plant samples will validate the utility of this rapid approach in on-site diagnosis.

The detection of phytoplasmas from insects is fundamental to the identification of potential insect vectors and in the study of their epidemiology. While detection has conventionally required DNA extraction from the whole body of the insect (49, 51), phytoplasmas accumulate within various organs including the alimentary canal, hemolymph, and salivary glands (52, 53). Thus, a recent study employed column-mediated DNA extraction from the legs, which contain hemolymph (54). In this study, the simple boiling extraction method led to the detection of phytoplasmas even from a single leg. In this way, tested individuals need not be sacrificed solely for extraction; they can be used, for example, as material for abdominal microinjection into healthy insects (55, 56) or for phytoplasma dynamics analysis in insects (54, 57). In conclusion, by simplifying and shortening the time for phytoplasma detection, the simple DNA extraction method and practical protocols increase both the speed and the efficiency of the entire CaPU23S-4 LAMP system.

In this study, by focusing on 23S rDNA, universal LAMP detection at the phytoplasma genus level was achieved. For LAMP primer design, it could utilize the full-length 23S rDNA sequences of phytoplasmas and the bacteria closely related to phytoplasmas, a considerable number of which have been provided by whole-genome sequencing (58–60). To date, 23S rDNA sequences have received little attention in bacterial research. However, with the accumulation of the whole-genome sequences of an increasing number of bacterial species, 23S rDNA may serve as a valuable gene target for the comprehensive detection of plant pathogenic bacteria. Plant disease diagnostics often extend to a detailed classification such as at the levels of species, subspecies, and genetic group, which can contribute to the discovery of new pathogens. However, many pathogens, including phytoplasmas, often share similar epidemiological characteristics, such as transmission patterns and habitats in plants and the environment, in closely related species, making it possible to establish measures to control the disease if they can be identified in a category larger than the species. The LAMP method for comprehensive detection of a plant pathogenic bacterial groups, focusing on 23S rDNA, can be a rapid and practical detection technique that is applicable to a variety of crop production fields. Such universal detection systems may offer a new direction in reducing the need to use individual pathogen detection techniques for the diagnosis of plant diseases and contribute to improving the efficiency of disease control.

## MATERIALS AND METHODS

### DNA samples

Plant DNAs infected with each of 28 ‘*Ca*. Phytoplasma’ species were used (Table S2). For three ‘*Ca*. Phytoplasma’ species whose total DNAs were not available from infected plants (‘*Ca*. P. australiense’, ‘*Ca*. P. pini’, and ‘*Ca*. P. luffae’), DNA fragments containing the target region of CaPU23S-4 were synthesized by GeneArt Gene Synthesis Service (Thermo Fisher Scientific, Waltham, MA, USA) based on the sequences deposited in the NCBI database (Fig. 1; Table S2). Other bacterial DNA samples are listed in Table S5. Purified genomic DNAs from species in the class Mollicutes (*Acholeplasma laidlawii*, *Spiroplasma citri*, *Mycoplasma genitalium*, and *Ureaplasma urealyticum*) were purchased (Minerva Biolabs GmbH, Berlin, Germany), and 100 fg from each one was subjected to the LAMP assay. Genomic DNAs of the following bacteria were extracted and purified using the CTAB method: *Bacillus subtilis*, *Clavibacter michiganensis*, *Agrobacterium tumefaciens*, *Acidovorax avenae*, *Burkholderia andropogonis*, *Ralstonia pseudosolanacearum*, *Pectobacterium carotovorum*, *Pseudomonas syringae*, *Xanthomonas campestris*, and *Escherichia coli*. Then, the LAMP assay was run on the samples. The healthy plant samples used are listed in Table S6. Total DNA was extracted from leaf midrib according to the CTAB method using GENE PREP STAR PI-80X (Kurabo, Osaka, Japan); then, the samples were subjected to PCR and LAMP assays.

### LAMP assay targeting 23S rDNA

The full-length 23S rDNA sequences were obtained from the NCBI online database: ‘*Ca*. P. asteris’ (NC005303), ‘*Ca*. P. mali’ (CU469464), ‘*Ca*. P. aurantifolia’ (JQ359013), ‘*Ca*. P. pruni’ (AF277076), ‘*Ca*. P. phoenicium’ (HM559253), ‘*Ca*. P. oryzae’ (CP116038), ‘*Ca*. P. palmae’ (FO681347), and ‘*Ca*. P. ziziphi’ (GU723425) (Fig. S1). Four candidate LAMP primers were designed using Primer explorer V4 software (http://primerexplorer.jp/) based on the 23S rDNA sequence of ‘*Ca*. P. asteris’ (NC005303). The LAMP reactions consisted of 20 mM Tris-HCl (pH 8.8), 10 mM KCl, 8 mM MgSO_4_, 10 mM (NH_4_)_2_SO_4_, 0.1% Tween 20, 0.8 M betaine, 1.4 mM of each dNTP, 8 U *Bst* DNA polymerase (New England Biolabs, Ipswich, MA, USA), 1.0 µL fluorescent detection reagent (FD, Eiken Chemical, Tokyo, Japan), and each of the primer sets (1.6 µM for FIP and BIP, 0.2 µM for F3 and B3, and 0.8 µM for LF and LB) in a 25 µL reaction volume. Following the addition of template DNA, the samples were incubated at the optimal temperature for each primer (64°C for CaPU23S-4) for 60–90 min to amplify the 23S rDNA. The reaction was stopped by incubation at 80°C for 2 min to inactivate DNA polymerase. The assays were performed using a Genie I (OptiGene Ltd. Horsham, UK) or a thermal cycler (Applied Biosystems ProFlex PCR System, Thermo Fisher Scientific). DNA amplification was detected according to colorimetric changes in the reaction mixture or changes in the fluorescence intensity under ultraviolet light. Data from the real-time LAMP assay were analyzed using Genie II software (OptiGene).

### Construction of a phylogenetic tree

The 16S rDNA sequences of the phytoplasmas and *A. laidlawii* obtained from the NCBI database were aligned with MUSCLE (MEGA7) and then phylogenetically analyzed according to the neighbor-joining method using MEGA7 (61) (Table S3). The outgroups were *A. laidlawii* BN1-JA1 (JN935888) and *B. subtilis* (NR112116).

### Alignment of the CaPU23S-4 target region in phytoplasmas and closely related bacteria

The CaPU23S-4 annealing region of each ‘*Ca*. Phytoplasma’ species was sequenced using two sets of PCR primers designed based on sequences outside of that region (Table S1). DNAs from the phytoplasma-infected plant samples (Table S2) were subjected to nested PCRs using two primer pairs (with some nucleotide mismatches): 23SP-1pf/23SP-1pr or 23SP-2pf/23SP-2pr for the first PCR and 23SP-1nf/23SP-1nr or 23SP-2nf/23SP-2nr for the second PCR. The products of the first PCR served as template DNAs for the second PCR after their 50-fold dilution. Both PCR amplifications were performed using KOD FX Neo (Toyobo, Osaka, Japan) under the following conditions: 94°C for 2 min, followed by 35 cycles of 98°C for 10 s, 55°C for 30 s, and 68°C for 2 min. The final extension was at 68°C for 7 min. Before Sanger sequencing (Eurofins Genomics, Edersberg, Germany), the nested PCR products were purified using ExoSAP-IT (Thermo Fisher Scientific) to remove excess nucleotides and primers. The obtained sequences of the 28 species were aligned with those of ‘*Ca*. P. australiense’, ‘*Ca*. P. pini’, and ‘*Ca*. P. luffae’ using the ATSQ software packaged in GENETYX-MAC Ver. 19 (GENETYX, Tokyo, Japan). In addition, the 23S rDNA sequences of the following bacteria were obtained from the NCBI database: *A. palmae* (FO681347), *A. brassicae* (FO681348), *A. laidlawii* (CP000896), *E. ellychniae* (PHND01000001), *M. mycoides* (CP010267), *M. genitalium* (NC000908), *M. pneumoniae* (CP008895), *Ms. florum* (CP006778), *U. urealyticum* (NR103078), *S. citri* (AM285316), and *B. subtilis* (AP011541). The CaPU23S-4 annealing regions of these bacteria and of the eight phytoplasma species from each of the 16S-groups were aligned using the ATSQ software (Fig. S1).

### Detection sensitivity of CaPU23S-4

Total DNA extracted from ‘*Ca*. P. asteris’-infected *G. coronaria* (experimental plant) by CTAB-based method was 10-fold serially diluted with total DNA from healthy plants to obtain concentrations ranging from 10 ng to 10 fg. The PCR for the universal detection of phytoplasmas was performed using P1/P7 primer set, which amplifies an approximately 1.8 kbp fragment including the 16S rDNA (34, 37). The PCR amplifications were performed using TaKaRa Taq (Takara Bio) under the following conditions: 94°C for 3 min, followed by 35 cycles of 94°C for 10 s, 55°C for 30 s, and 72°C for 2 min. The final extension was at 72°C for 7 min. PCR products were electrophoresed on 0.7% agarose gels and detected via ethidium bromide staining. LAMP assay using CaPU23S-4 was performed under the conditions described above.

### Boiling extraction method for on-site detection

A leaf midrib (5 × 2 mm) or an insect body was dipped into 100 µL STE buffer (100 mM NaCl, 10 mM Tris-HCl [pH 8.0], 1 mM EDTA) and incubated at 95°C for 10 min; 1.0 µL supernatant was used in the LAMP assay. However, in the case of coconut palm (*C. nucifera*), 50 mg of trunk sawdust was dipped into 1 mL STE buffer, and in the case of insect leg, a single hind leg or alimentary canal from each insect was dipped into 10 µL STE buffer. Experimental plants (*C. roseus*, *G. coronaria*), field plants (*C. nucifera*, *E. zollingeri*, *H. macrophylla*, *M. esculenta*, and *Musa* sp.) and laboratory-maintained insect vector (*M. striifrons*) were subjected to LAMP assay in the laboratory. Infected insect samples were prepared by allowing *M. striifrons* to feed on ‘*Ca*. P. asteris’-infected *G. coronaria* for 28 days. For the boiling extraction of *E. zollingeri*, a micro-spatula was used to add PVPP (Sigma Aldrich) to 100 µL STE buffer in amounts consistent with the method of Hanaoka and Fukuda (62). DNA was also extracted according to the CTAB-based method using GENE PREP STAR PI-80X (KURABO) and according to the Whatman FTA card method (Whatman Inc., Florham Park, NJ, USA). For insect samples, DNA was extracted using the DNeasy blood and tissue kit (QIAGEN, Hilden, Germany) instead of the CTAB-based method. After either one, 10 ng purified DNA was used in the LAMP assay. In the FTA method, DNA was captured and washed on the FTA card as described in the manufacturer’s protocol, with one disk (Φ2 mm) directly added to the reaction mixture of the LAMP assay.

## Data Availability

All partial 23S rDNA sequences of the 27 phytoplasma species newly determined in this study were submitted to DDBJ under accession numbers LC790974, LC790975, LC790976, LC790977, LC790978, LC790979, LC790980, LC790981, LC790982, LC790983, LC790984, LC790985, LC790986, LC790987, LC790988, LC790989, LC790990, LC790991, LC790992, LC790993, LC790994, LC790995, LC790996, LC790997, LC790998, LC790999, and LC791000 (Table S2).

## SUPPLEMENTAL MATERIAL

Supplemental material spectrum.00106-24-s0001.pdf Tables S1 to S7; Fig. S1 to S6.

ASM does not own the copyrights to Supplemental Material that may be linked to, or accessed through, an article. The authors have granted ASM a non-exclusive, world-wide license to publish the Supplemental Material files. Please contact the corresponding author directly for reuse.
