# Supplementary material for: Use of the 23S rRNA gene as a target template in the universal loop-mediated isothermal amplification (LAMP) of genomic DNA from phytoplasmas
Source: Microbiol Spectr. 2024 Mar 27;12(5):e00106-24. doi: 10.1128/spectrum.00106-24 (PMC11064480; doi:10.1128/spectrum.00106-24)
Supplement: Supplemental material — Tables S1 to S7; Fig. S1 to S6. [file spectrum.00106-24-s0001.pdf]

Supplementary material

Supplementary table

Table S1 Primer sets designed for this study

Objective	Primer name	Sequence (5'-3')	Remarks
LAMP	CaPU23S-1-F3	CGGAAGCAGTGGTGGAGA	Optimal temperature: 65°C
	CaPU23S-1-B3	TGTCCCTCCGTCAGTTGAT	
	CaPU23S-1-FIP	GGCCCTGGAAACCTTGGGTTTTGGTCAGAAGTGAGAATGCCG	
	CaPU23S-1-BIP	TTCGTCCTCCCTGGGTTAGTCGAGTGCAGGAATCTCAGCCT	
	CaPU23S-1-LF	ACGGATAGGATTCTCACCTATCTTT	
	CaPU23S-1-LB	AGGGAGAAATCCGTAATCGATGGCA	
	CaPU23S-2-F3	GGAGCTTGACTGCAAGACT	Optimal temperature: 64°C
	CaPU23S-2-B3	TCCAGGATGCGATGAGCC	
	CaPU23S-2-FIP	CCGTTGAGTCACGGCCCTTCGCAGGGACGAAAGTCGGA	
	CaPU23S-2-BIP	AGCTACCCTGGGGATAACAGGCCATCGAGGTGCCAAACCG	
	CaPU23S-2-LF	CATTCGGTACCGTAAGATCACTAAG	
	CaPU23S-2-LB	CGCTTCCAAGCGTTCACAGC	
	CaPU23S-3-F3	TTTGGCACCTCGATGTCG	Optimal temperature: 63°C
	CaPU23S-3-B3	CAACTGAAACACCAGCGGTA	
	CaPU23S-3-FIP	TCCGCTTTAATGGGCGAACAGCCTCATCGCATCCTGGAGCT	
	CaPU23S-3-BIP	TTCGGTCTCTATCCGTCGTGGGATCCATCCCGGTCTTCTCA	
	CaPU23S-3-LF	CCAACCCTTGGAACCTTCTCC	
	CaPU23S-3-LB	TTGGAAATTTGAAAGGAACTGTCCC	
PCR	23SP-1pf	CTAAARAGTAACGGAGGCGTTC	Used for sequence analysis of 16 phytoplasmas shown in Fig. S4a
	23SP-1pr	CACCTCTRGCCTATCAACCTG	
	23SP-1nf	CGAAAGTCGGACTTAGTGATC	
	23SP-1nr	GTCTTTCAGGGAYCTCATAGG	
	23SP-2pf	AAAGAGTAACGGAGGCGTTC	Used for sequence analysis of 11 phytoplasmas shown in Fig. S4b
	23SP-2pr	TCACTCTTCTTACACCTCTAG	
	23SP-2nf	GTCGGACTTAGTGATCTTAC	
	23SP-2nr	TCTTTCAGGGACCTCATAGG	

Table S2 '*Candidatus* Phytoplasma' species used in this study

16S-group[a]	'*Ca.* Phytoplasma' species	Strain	Disease	Source	Accession[c]
i	'*Ca.* P. asteris' *	OY	Onion yellows	Our lab.	NC005303
	'*Ca.* P. australiense'[b]	-	-	-	NR076245
	'*Ca.* P. japonicum'	JHP	Japanese hydrangea phyllody	Our lab.	LC790974
	'*Ca.* P. fragariae'	SY	Strawberry yellows	Chiba Prefectural Agriculture and Forestry Research Center (Japan)	LC790975
	'*Ca.* P. solani'	STOL	Stolbur	A. Bertaccini (University of Bologna, Italy)	LC790976
	'*Ca.* P. convolvuli'	-	-	A. Bertaccini	LC790977
	'*Ca.* P. meliae'	-	-	A. Bertaccini	LC790978
ii	'*Ca.* P. mali' *	AP	Apple proliferation	A. Bertaccini	LC790979
	'*Ca.* P. pyri'	PD	Pear decline	A. Bertaccini	LC790980
	'*Ca.* P. prunorum'	LNS	Plum leptonecrosis	A. Bertaccini	LC790981
	'*Ca.* P. tamaricis'	SCWB	Salt cedar witches'-broom	W. wei (Beltsville Agricultural Research Center, USA)	LC790982
iii	'*Ca.* P. aurantifolia' *	WBDL	Witches'-broom of lime	A. Bertaccini	LC790983
	'*Ca.* P. brasiliense'	SuV	Surinam virescence	A. Bertaccini	LC790984
iv	'*Ca.* P. pruni' *	TWB	Tsuwabuki witches'-broom	Our lab.	LC790985
v	'*Ca.* P. phoenicium' *	NaxY	Naxos yellows	A. Bertaccini	LC790986
	'*Ca.* P. omanense'	CWB	Cassia witches'-broom	A. Bertaccini	LC790987
vi	'*Ca.* P. palmae'	LY	Coconut lethal yellowing	A. Bertaccini	LC790988
	'*Ca.* P. castaneae'	CnWB	Chestnut witches'-broom	Our lab.	LC790989
	'*Ca.* P. pini'[b]	MDPP	Witches'-broom of pinus	-	KU242428
	'*Ca.* P. palmicola'	-	-	A. Bertaccini	LC790990
	'*Ca.* P. noviguineense' *	BW	Banana wilt	Our lab.	LC790991
vii	'*Ca.* P. oryzae' *	RYD	Rice yellow dwarf	Our lab.	LC790992
	'*Ca.* P. cynodontis'	BGWL	Bermuda grass white leaf	A. Bertaccini	LC790993
viii	'*Ca.* P. ulmi'	EY	Elm yellows	A. Bertaccini	LC790994
	'*Ca.* P. vitis'	ALY	Alder yellows	A. Bertaccini	LC790995
	'*Ca.* P. ziziphi' *	JWB	Jujube witches'-broom	Our lab.	LC790996
	'*Ca.* P. rubi'	RS	Rubus stunt	A. Bertaccini	LC790997
	'*Ca.* P. trifolii'	CP	Clover proliferation	A. Bertaccini	LC790998
	'*Ca.* P. fraxini'	AshY	Ash yellows	A. Bertaccini	LC790999
	'*Ca.* P. luffae'[b]	LfWB	Loofah witches'-broom	-	AF086621
	'*Ca.* P. malaysianum'	ELY	Elaeocarpus yellows	M. Satoh (University of Tokushima, Japan)	LC791000

[a] Previously reported phylogenetic group names (26, 27).

[b] Artificially synthesized DNA based on 23S rDNA sequence in NCBI database were used.

[c] Sequences of the CaPU23S-4 amplified region on 23S rDNA used in this study.

* Eight phytoplasma species used for selection of universal LAMP primers.

Table S3 16S rDNA sequence of phytoplasmas used for phylogenetic tree construction

16S-group[a]	'*Ca.* Phytoplasma' species	Accession
i	'*Ca.* P. asteris'	M30790
	'*Ca.* P. lycopersici'	EF199549
	'*Ca.* P. tritici'	DQ078304
	'*Ca.* P. australiense'	L76865
	'*Ca.* P. japonicum'	AB010425
	'*Ca.* P. fragariae'	HM104662
	'*Ca.* P. solani'	AF248959
	'*Ca.* P. convolvuli'	JN833705
	'*Ca.* P. hispanicum'	AF248960
	'*Ca.* P. meliae'	KU850940
	'*Ca.* P. graminis'	AY725228
	'*Ca.* P. caricae'	AY725234
	'*Ca.* P. americanum'	DQ174122
	'*Ca.* P. costaricanum'	HQ225630
ii	'*Ca.* P. mali'	AJ542541
	'*Ca.* P. pyri'	AJ542543
	'*Ca.* P. prunorum'	AJ542545
	'*Ca.* P. spartii'	X92869
	'*Ca.* P. rhamni'	X76431
	'*Ca.* P. tamaricis'	FJ432664
	'*Ca.* P. allocasuarinae'	AY135523
iii	'*Ca.* P. aurantifolia'	U15442
	'*Ca.* P. australasia'	Y10097
	'*Ca.* P. brasiliense'	AF147708
iv	'*Ca.* P. pruni'	JQ044397
v	'*Ca.* P. phoenicium'	AF515636
	'*Ca.* P. omanense'	EF666051
vi	'*Ca.* P. palmae'	U18747
	'*Ca.* P. cocostanzaniae'	X80117
	'*Ca.* P. castaneae'	AB054986
	'*Ca.* P. pini'	AJ632155
	'*Ca.* P. palmicola'	KF364359
	'*Ca.* P. noviguineense'	LC228755
	'*Ca.* P. dypsidis'	MT536195
vii	'*Ca.* P. oryzae'	D12581
	'*Ca.* P. cirsii'	KR869146
	'*Ca.* P. sacchari'	MN889545
	'*Ca.* P. cynodontis'	AJ550984
	'*Ca.* P. wodyetiae'	KC844879
viii	'*Ca.* P. ulmi'	AY197655
	'*Ca.* P. vitis'	AF176319
	'*Ca.* P. ziziphi'	AB052876
	'*Ca.* P. rubi'	AY197648
	'*Ca.* P. balanitae'	AB689678
	'*Ca.* P. trifolii'	AY390261
	'*Ca.* P. sudamericanum'	GU292081
	'*Ca.* P. fraxini'	AF092209
	'*Ca.* P. luffae'	AF248956
	'*Ca.* P. malaysianum'	EU371934
	'*Ca.* P. stylosanthis'	MT431550

[a] Previously reported phylogenetic group names (26, 27).

Table S4 Summary of detectable range of each LAMP primer in phytoplasma detection

16S-group[a]	'*Ca.* Phytoplasma' species	16S rRNA RFLP group	LAMP amplification			
			This study	Obura et al. (63)	Dickinson (64), Quoc et al. (65)	Yu et al. (66)
i	'*Ca.* P. asteris'	16SrI	+	NT	+	+
	'*Ca.* P. lycopersici'		NT			
	'*Ca.* P. tritici'		NT			
	'*Ca.* P. australiense'	16SrXII	+	+	NT	NT
	'*Ca.* P. japonicum'		+			
	'*Ca.* P. fragariae'		+			
	'*Ca.* P. solani'		+			
	'*Ca.* P. convolvuli'		+			
	'*Ca.* P. hispanicum'	16SrXIII	NT	NT	NT	NT
	'*Ca.* P. meliae'		+			
	'*Ca.* P. graminis'	16SrXVI	NT	NT	NT	NT
	'*Ca.* P. caricae'	16SrXVII	NT	NT	NT	NT
	'*Ca.* P. americanum'	16SrXVIII	NT	NT	NT	NT
	'*Ca.* P. costaricanum'	16SrXXXI	NT	NT	NT	NT
ii	'*Ca.* P. mali'	16SrX	+	+	+	NT
	'*Ca.* P. pyri'		+			
	'*Ca.* P. prunorum'		+			
	'*Ca.* P. spartii'		NT			
	'*Ca.* P. rhamni'	16SrXX	NT	NT	NT	NT
	'*Ca.* P. tamaricis'	16SrXXX	+	NT	NT	NT
	'*Ca.* P. allocasuarinae'	16SrXXXIII	NT	NT	NT	NT
iii	'*Ca.* P. aurantifolia'	16SrII	+	NT	+	NT
	'*Ca.* P. australasia'		NT			
	'*Ca.* P. brasiliense'	16SrXV	+	NT	NT	NT
iv	'*Ca.* P. pruni'	16SrIII	+	NT	+	NT
v	'*Ca.* P. phoenicium'	16SrIX	+	NT	NT	NT
	'*Ca.* P. omanense'	16SrXXIX	+	NT	NT	NT
vi	'*Ca.* P. palmae'	16SrIV	+	NT	+	+
	'*Ca.* P. cocostanzaniae'		NT			
	'*Ca.* P. castaneae'	16SrXIX	+	NT	NT	NT
	'*Ca.* P. pini'	16SrXXI	+	NT	NT	NT
	'*Ca.* P. palmicola'	16SrXXII	+	NT	+	+
	'*Ca.* P. noviguineense'	16SrXXXVIII	+	NT	NT	NT
	'*Ca.* P. dypsidis'	16SrXXXIX	NT	NT	NT	NT
vii	'*Ca.* P. oryzae'	16SrXI	+	+	+	+
	'*Ca.* P. cirsii'		NT			
	'*Ca.* P. sacchari'		NT			
	'*Ca.* P. cynodontis'	16SrXIV	+	+	NT	+
	'*Ca.* P. wodyetiae'	16SrXXXVI	NT	NT	NT	NT
viii	'*Ca.* P. ulmi'	16SrV	+	NT	NT	NT
	'*Ca.* P. vitis'		+			
	'*Ca.* P. ziziphi'		+			
	'*Ca.* P. rubi'		+			
	'*Ca.* P. balanitae'		NT			
	'*Ca.* P. trifolii'	16SrVI	+	+	+	NT
	'*Ca.* P. sudamericanum'		NT			
	'*Ca.* P. fraxini'	16SrVII	+	NT	NT	NT
	'*Ca.* P. luffae'	16SrVIII	+	NT	NT	NT
	'*Ca.* P. malaysianum'	16SrXXXII	+	NT	NT	NT
	'*Ca.* P. stylosanthis'	16SrXXXVII	NT	NT	NT	NT
		Total 31 groups	22 groups	5 groups	8 groups	5 groups

[a] Previously reported phylogenetic group names (26, 27).

Table S5 Cross-reactivity of the phytoplasma LAMP assay with other bacteria

Phylum	Class	Species	Strain/Isolate	Strain no.	DNA volume (ng / test) [a]	LAMP amplification	
						60 min	90 min
Firmicutes	Mollicutes	*Acholeplasma laidlawii*	PG8	ATCC23206	0.1	-	- [b]
		Mycoplasma genitalium	G37	ATCC33530	0.1	-	-
		Spiroplasma citri	Morocco-R8-A2	ATCC27556	0.1	-	-
		Ureaplasma urealyticum	T960	ATCC27618	0.1	-	-
	Bacilli	*Bacillus subtilis*	Marburg	ATCC6051-U	10	-	-
Actinomycetota	Actinomycetia	*Clavibacter michiganensis* subsp. *michiganensis*	N6301	MAFF301040	10	-	-
Pseudomonadota	Alphaproteobacteria	*Agrobacterium tumefaciens*	-	MAFF 301001	10	-	-
	Betaproteobacteria	*Acidovorax avenae* subsp. *avenae*	H-8201	MAFF301502	10	-	-
		Burkholderia andropogonis	76E-1	MAFF301116	10	-	-
		Ralstonia pseudosolanacearum	Tt7322	MAFF211266	10	-	-
	Gammaproteobacteria	*Escherichia coli*	DH5 alpha	-	10	-	-
		Pectobacterium carotovorum subsp. *carotovorum*	E7116	MAFF301394	10	-	-
		Pseudomonas syringae pv. *tomato*	DC3000	NCPPB4369	10	-	-
		Xanthomonas campestris pv. *campestris*	-	ATCC33913	10	-	-

[a] Purified genomic DNA of each bacteria species.

[b] LAMP amplification was observed at about 70 minutes in one of the four replicates in *A. laidlawii* .

Table S6 Cross-reactivity of the phytoplasma LAMP assay with host plants

Eudicots / Monocots	Family	Species	Name	DNA volume (ng / test) [a]	LAMP amplification	
					60 min	90 min
Eudicots	Apocynaceae	*Catharanthus roseus*	Periwinkle	10	-	-
	Asteraceae	*Chrysanthemum coronarium*	Garland chrysanthemum	10	-	-
	Elaeocarpaceae	*Elaeocarpus zollingeri*	Elaeocarpus	10	-	-
	Euphorbiaceae	*Manihot esculenta*	Cassava	10	-	-
	Fabaceae	*Glycine max*	Soybean	10	-	-
	Hydrangeaceae	*Hydrangea macrophylla*	Hydrangea	10	-	-
	Paulowniaceae	*Paulownia tomentosa*	Paulownia	10	-	-
	Rhamnaceae	*Ziziphus jujuba*	Jujube	10	-	-
	Rosaceae	*Fragaria* × *ananassa*	Strawberry	10	-	-
		Malus domestica	Apple	10	-	-
		Prunus persica	Peach	10	-	-
	Solanaceae	*Solanum lycopersicum*	Tomato	10	-	-
		Solanum tuberosum	Potato	10	-	-
	Vitaceae	*Vitis* sp.	Grapevine	10	-	-
Momocots	Amaryllidaceae	*Allium cepa*	Onion	10	-	-
	Arecaceae	*Cocos nucifera*	Coconut palm	10	-	-
	Poaceae	*Oryza sativa*	Rice	10	-	-

[a] Total DNAs were extracted from leaf vein of each healthy plant by CTAB method.

Table S7 Optimization of boiling extraction protocol for coconut palm sawdust

Sawdust:STE buffer ratio in boiling extraction (Actual amount)		LAMP result (Positive / Total)		
		Healthy sample	Infected sample	
			+++[a]	+
1:10	(10 mg:100 μL)	0/3	1/3	NT
1:20	(50 mg:1 mL)	0/3	3/3	4/4
1:100	(10 mg:1 mL)	0/3	3/3	3/4
1:200	(5 mg:1 mL)	0/3	3/3	2/4

[a] The number of + indicates the level of phytoplasma accumulation, which was judged by PCR (P1/P7 primer set) from the same sawdust lots.

NT: not tested.

Supplementary figure

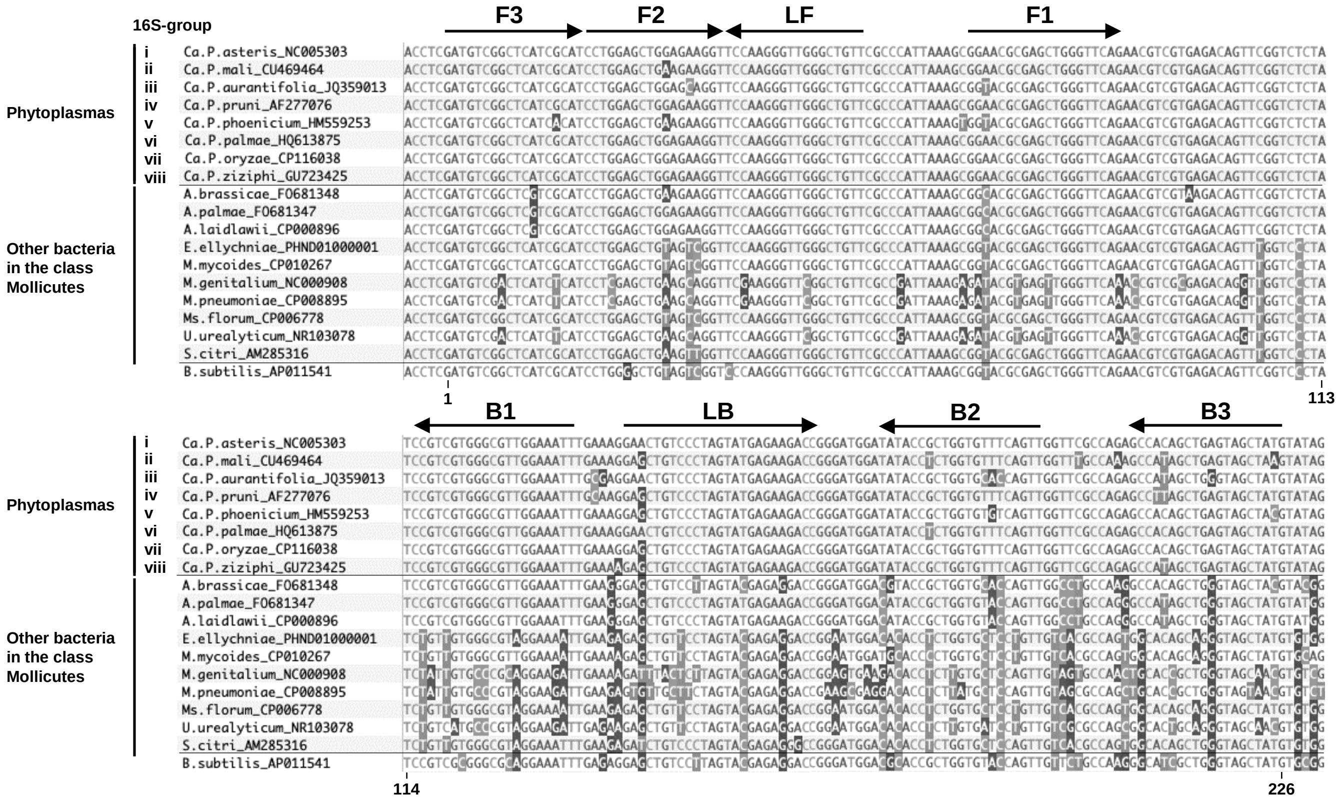

Fig. S1
Alignment of the CaPU23S-4 target region of phytoplasmas and other closely related bacterial species based on the sequences obtained from the NCBI database. Eight 16S-groups (i–viii) (26, 27) are shown in the left of phytoplasma species names. The positions and directions of the CaPU23S-4 primer designed in this study are indicated by arrows. Nucleotides that differ from CaPU23S-4 primer are highlighted.

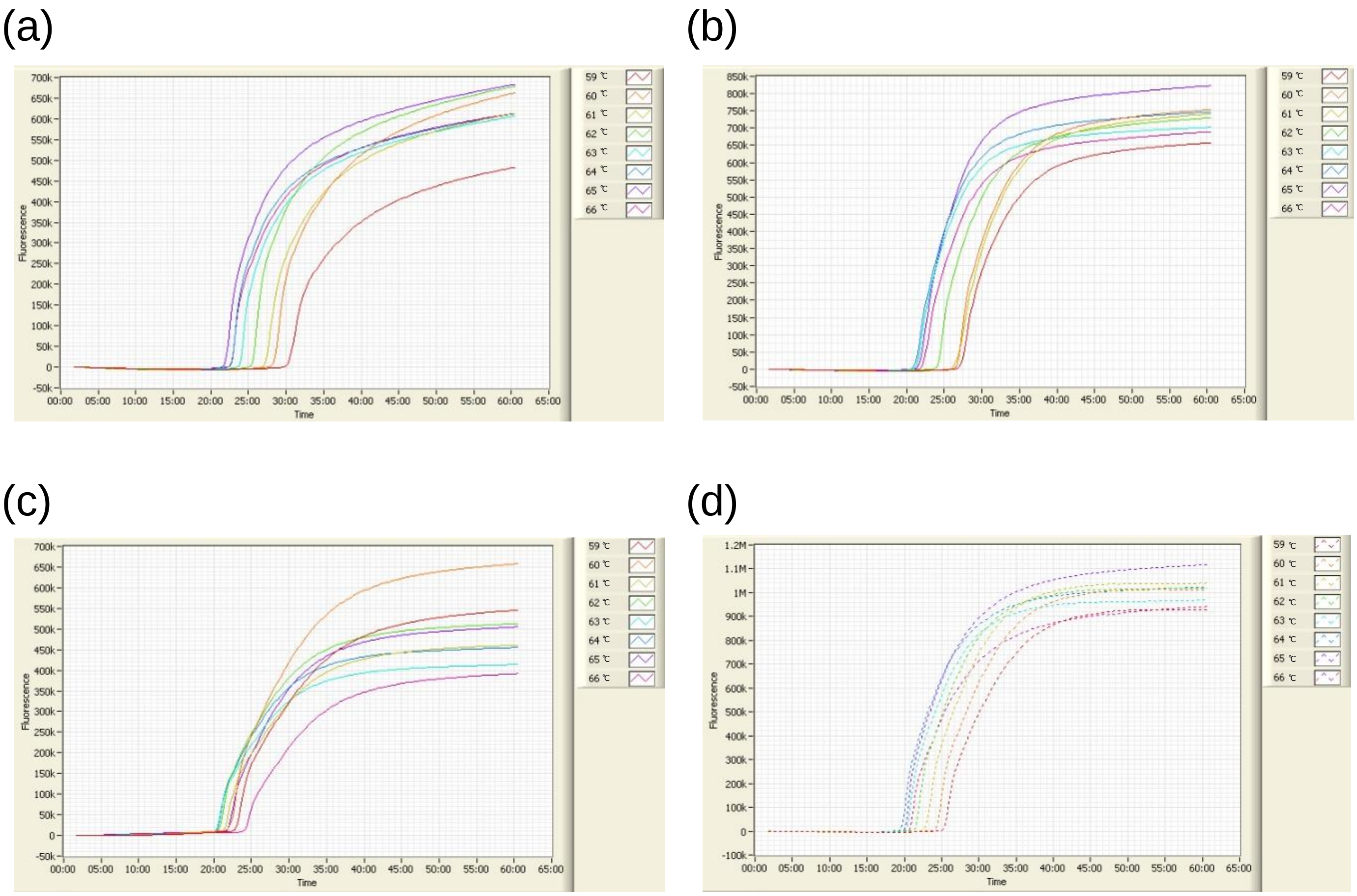

Fig. S2

Optimization of LAMP reaction temperature for the four primer sets designed in this study. LAMP amplification curves with CaPU23S-1 (a), CaPU23S-2 (b), CaPU23S-3 (c), CaPU23S-4 (d) primer sets are shown (Tables 1, S1). The LAMP reaction was performed at incubation temperatures of 59–66° C for 60 min. In all assays, 10 ng total DNA extracted from ‘*Ca.* P. asteris’-infected *C. coronarium* served as a template.

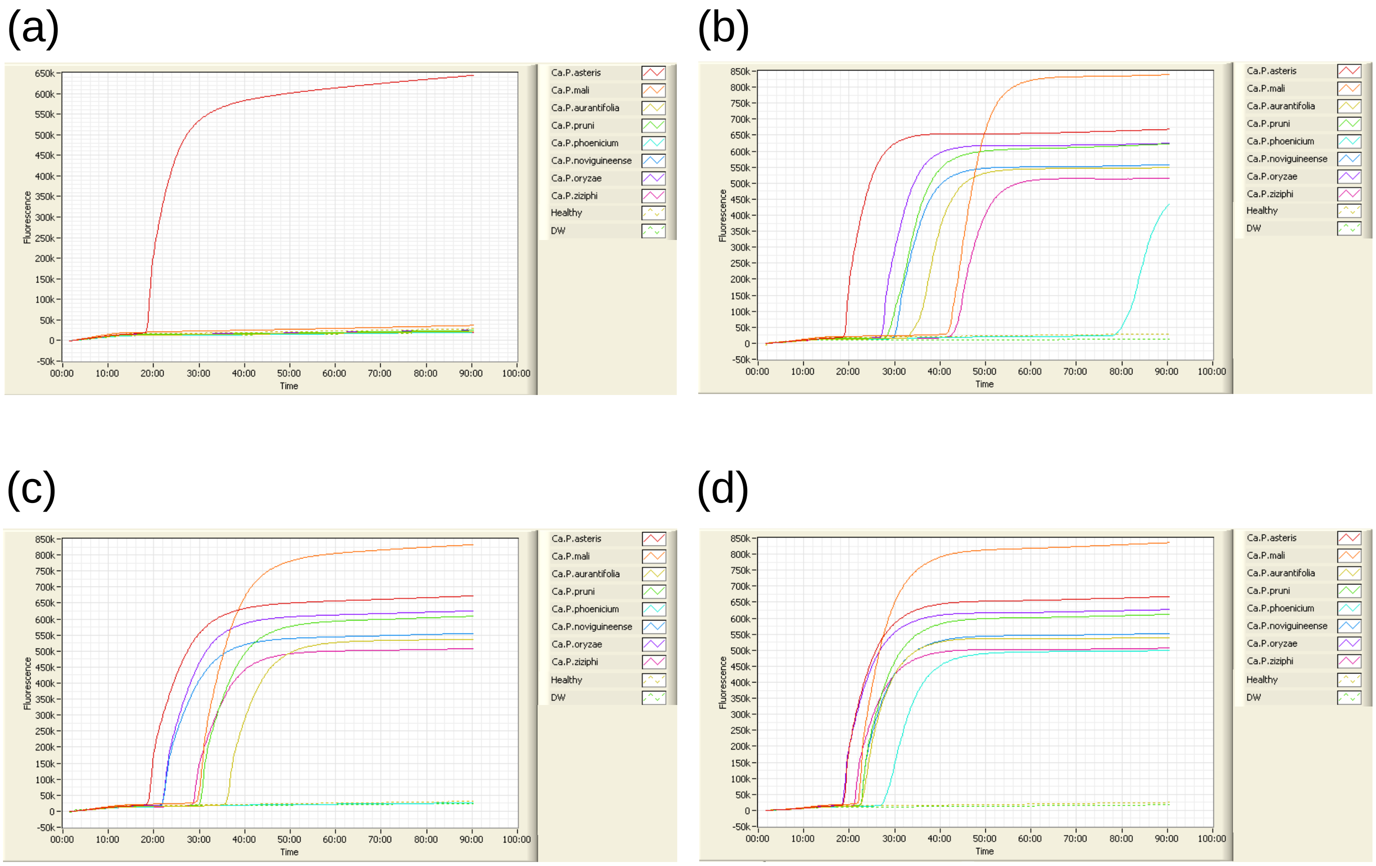

Fig. S3

Selection of the best LAMP primer sets for the universal detection of phytoplasmas. LAMP amplification curves with CaPU23S-1 (a), CaPU23S-2 (b), CaPU23S-3 (c), CaPU23S-4 (d) primer sets are shown (Tables 1, S1). Total DNA (10 ng each) from eight phytoplasma species covering all eight 16S-groups served as templates. The LAMP reactions were performed at the optimal temperature for each primer set (Table S1 and 64° C for CaPU23S-4) for 90 min. Healthy: negative control using total DNA (10 ng) from healthy *C. coronarium* as a template; DW: negative control using distilled water.

(a)

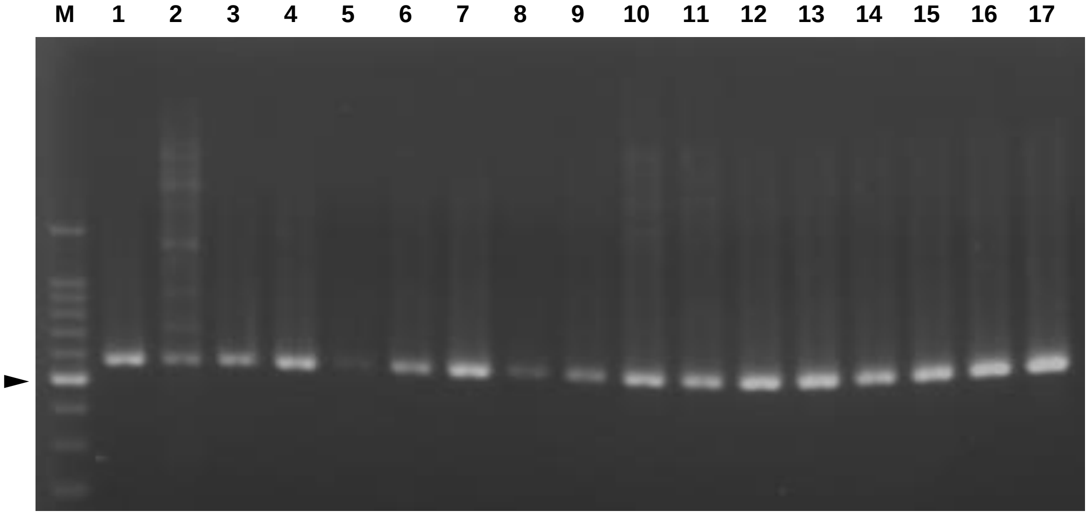

(b)

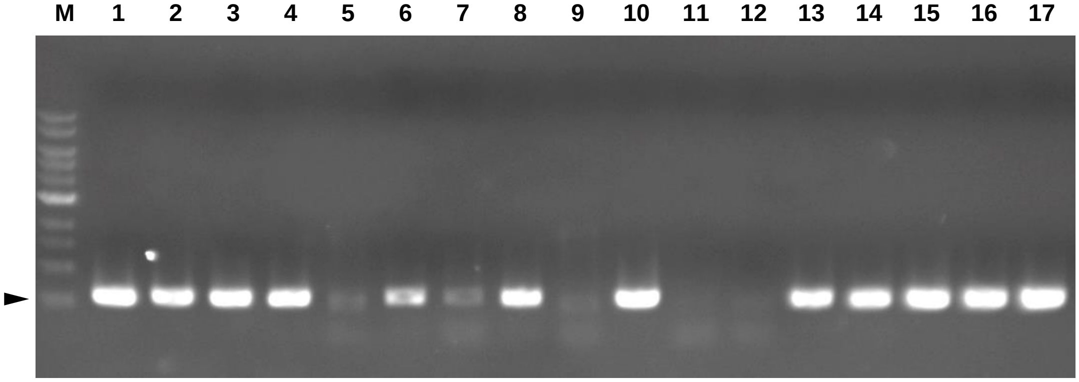

Fig. S4

PCR amplification of partial 23S rDNA. (a) Total DNAs (10 ng each) of 17 phytoplasmas were amplified using the 23SP-1pf/23SP-1pr primer set. Lanes 1: ‘*Ca*. P. fragariae’, 2: ‘*Ca*. P. convolvuli’, 3: ‘*Ca*. P. meliae’, 4: ‘*Ca*. P. pyri’, 5: ‘*Ca*. P. tamaricis’, 6: ‘*Ca*. P. aurantifolia’, 7: ‘*Ca*. P. brasiliense’, 8: ‘*Ca*. P. phoenicium’, 9: ‘*Ca*. P. omanense’, 10: ‘*Ca*. P. palmae’, 11: ‘*Ca*. P. palmicola’, 12: ‘*Ca*. P. noviguineense’, 13: ‘*Ca*. P. oryzae’, 14: ‘*Ca*. P. cynodontis’, 15: ‘*Ca*. P. rubi’, 16: ‘*Ca*. P. fraxini’, and 17: ‘*Ca*. P. asteris’ (as positive control). Lane M: 100 bp ladder markers. The PCR products were separated on a 2.0% agarose gel. Arrowhead indicates 500 bp. (b) Total DNAs (10 ng each) of 17 phytoplasmas were amplified using the 23SP-2pf/23SP-2pr primer set. Lanes 1: ‘*Ca*. P. vitis’, 2: ‘*Ca*. P. ulmi’, 3: ‘*Ca*. P. ziziphi’, 4: ‘*Ca*. P. trifolii’, 5: ‘*Ca*. P. fraxini’, 6: ‘*Ca*. P. malaysianum’, 7: ‘*Ca*. P. oryzae’, 8: ‘*Ca*. P. castaneae’, 9: ‘*Ca*. P. phoenicium’, 10: ‘*Ca*. P. pruni’, 11: ‘*Ca*. P. brasiliense’, 12: ‘*Ca*. P. aurantifolia’, 13: ‘*Ca*. P. prunorum’, 14: ‘*Ca*. P. mali’, 15: ‘*Ca*. P. asteris’ (as positive control), 16: ‘*Ca*. P. japonicum’, and 17: ‘*Ca*. P. solani’. Lane M: 1 kp ladder markers. The PCR products were separated on a 0.7% agarose gel. Arrowhead indicates 500 bp.

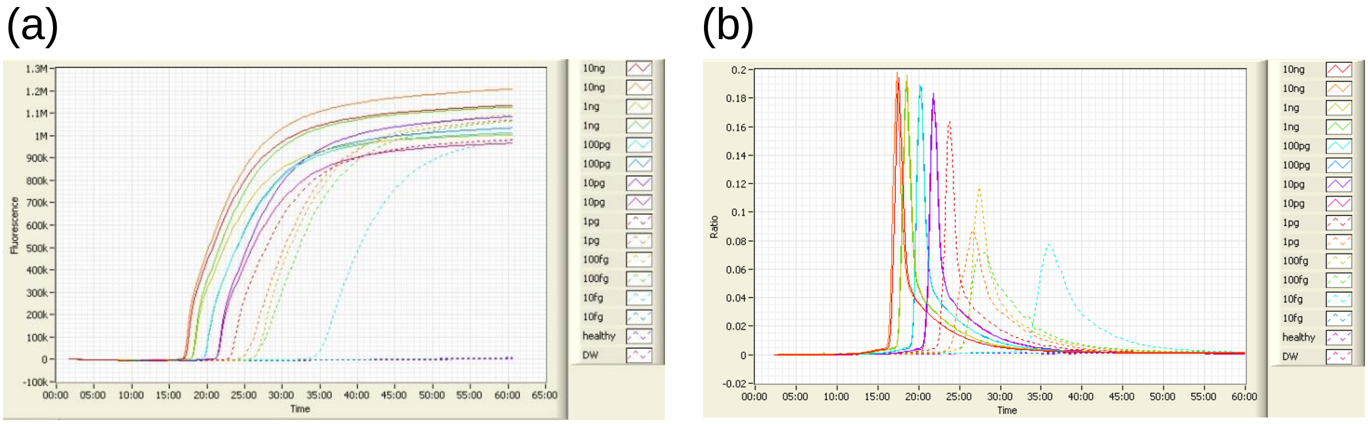

Fig. S5

Relationship between detection sensitivity and detection time for CaPU23S-4 LAMP assay. LAMP amplification curves (a) and fluorescence increasing ratio of LAMP amplification (b) of Fig. 3 were shown. 10-fold dilution series (ranging from 10 ng to10 fg per assay) of total DNA extracted from '*Ca*. P. asteris'-infected *C. coronarium* were used for each reaction. LAMP reaction was performed for 60 min at 64 ºC. Healthy: negative control using total DNA (10 ng) of healthy *C. coronarium* as a template, DW: negative control using distilled water.

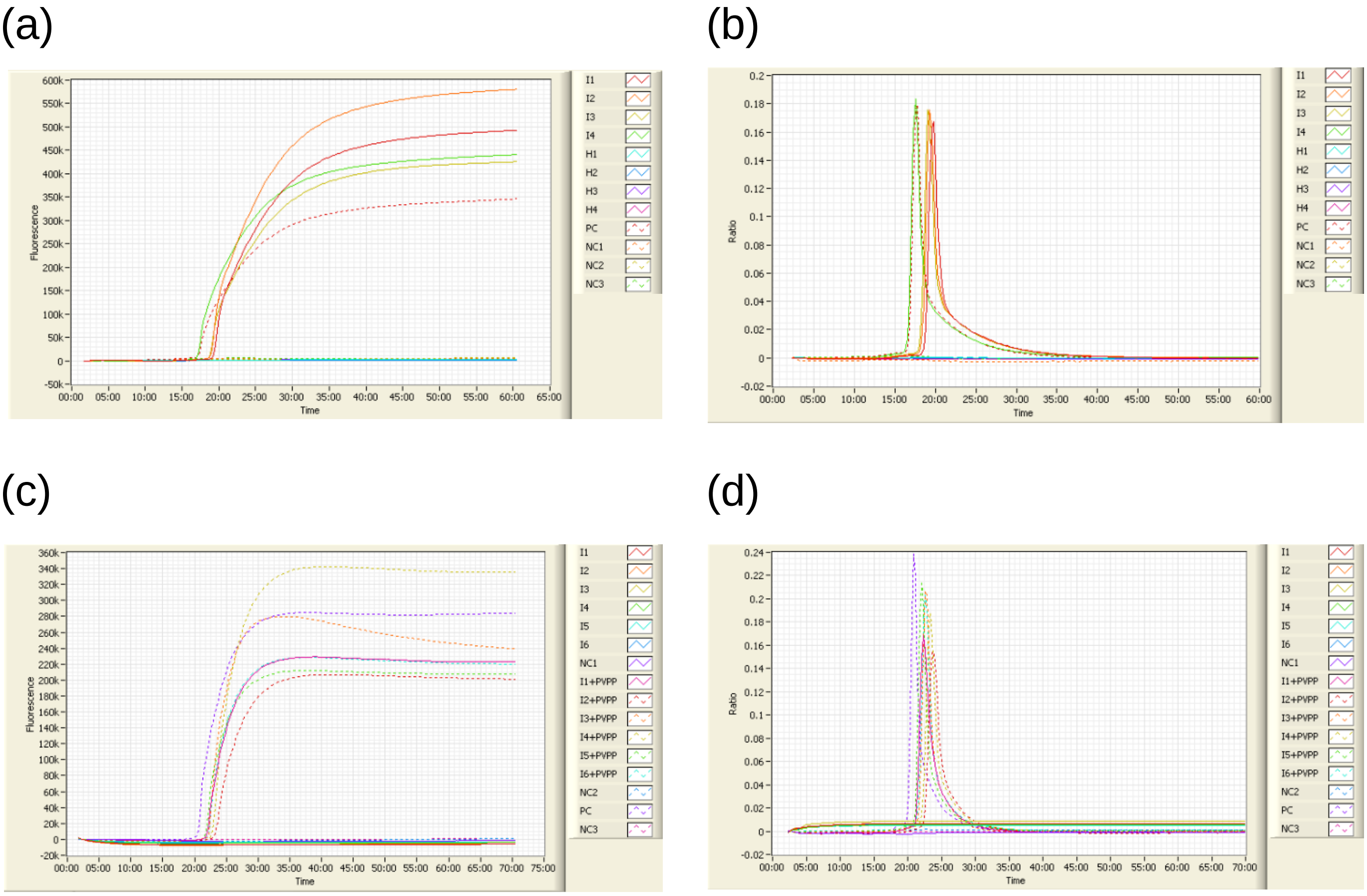

Fig. S6

Detection time of CaPU23S-4 LAMP assay in combination with boiling DNA extraction. LAMP amplification curves (a, c) and fluorescence increasing ratio of LAMP amplification (b, d) were shown. (a) DNAs extracted by boiling extraction from ‘*Ca*. P. asteris’-infected (I1–I4) and healthy (H1–H4) *C. coronarium* were used as templates. PC and NC1: positive and negative controls using total DNA (10 ng) of infected and healthy *C. coronarium*, respectively, extracted by CTAB-based method. NC2: negative control using STE buffer, NC3: negative control using distilled water. LAMP reaction was performed for 60 min at 64 ºC. (b) DNAs extracted by boiling extraction from ‘*Ca*. P. malaysianum’-infected *E. zollingeri* (I1–I6) and DNAs extracted by boiling extraction with PVPP (I1 + PVPP–I6 + PVPP) were used as templates. PC: positive control using total DNA (10 ng) of infected *E. zollingeri* extracted by CTAB-based method, NC1: negative control using STE buffer, NC2: negative control using STE buffer with PVPP, NC3: negative control using distilled water. LAMP reaction was performed for 70 min at 64 ºC.

Reference mentioned in supplementary data

63. Obura E, Masiga D, Wachira F, Gurja B, Khan ZR. 2011. Detection of phytoplasma by loop-mediated isothermal amplification of DNA (LAMP). J Microbiol Methods 84: 312–316. https://doi.org/10.1016/j.mimet.2010.12.011.

64. Dickinson M. 2015. Loop-mediated isothermal amplification (LAMP) for detection of phytoplasmas in the field, p 99–111. *In* Lacomme C (ed), Plant pathology. Methods in molecular biology, vol 1302. Humana Press, New York, NY. https://doi.org/10.1007/978-1-4939-2620-6_8.

65. Quoc NB, Xuan NTT, Phuong NDN, Trang HTT, Chau NNB , Duong CA, Dickinson M. 2021. Development of loop mediated isothermal amplification assays for the detection of sugarcane white leaf disease. Physiol Mol Plant Pathol 113: 101595. https://doi.org/10.1016/j.pmpp.2020.101595.

66. Yu S, Pan Y, Zhu H, Song W. 2023. Universal, rapid, and visual detection methods for phytoplasmas associated with coconut lethal yellowing diseases targeting 16S rRNA gene sequences. Plant Dis 107: 276–280. https://doi.org/10.1094/PDIS-05-22-0996-SC.
